# Frequency of VanA, VanB and VanH variants amongst vancomycin-resistant enterococci isolated from patients in central region of Iran

**Published:** 2016

**Authors:** Jaafar Rezvani, Reza Nasr, Fatemeh T. Shamsabadi, Mohammad Reza Akbari Eidgahi

**Affiliations:** 1 *Islamic Azad University, Damghn Branch, Damghan, Iran*; 2 *Semnan Biotechnology Research Center, Semnan University of Medical Sciences, Semnan, Iran *; 3 *Student Research Committee, Semnan University of Medical Sciences, Semnan, Iran*

**Keywords:** Vancomycin-resistance enterococci (VRE), *E. faecalis*, *E. faecium*, *vanA*, *vanH*, *vanB*, Patients

## Abstract

**Aim:**

The aim of this study was to investigate the VRE frequency and the rate of each gene in isolated enterococci from patients with intestinal infection in the central region of Iran.

**Background:**

*Enterococci* infections are a public health growing concern due to the glycopeptide antibiotics resistance especially vancomycin. Genes, *vanA*, *B*, and *H* contribute to the influence of vancomycin-resistant enterococci (VRE).

**Patients and methods:**

This study was conducted from January to July 2014 in Shahrood university hospital. Enterococci isolation and its antibacterial susceptibility were performed by culturing in Aesculin Azide agar and Kirby-Bauer method, respectively. Vancomycin-resistant genes were screened through conventional PCR, and subsequently sequenced.

**Results:**

Among 265 specimens, 100 isolates revealed enterococci, in which *E. faecalis* (91%) and *E. faecium* (9%). The isolated enterococci were resistant to vancomycin (6%) and chloramphenicol (21%), whereas their large proportions (94% to 100%) were multi-drug resistant. All VRE isolates belonged to *E. faecalis, *conversely, the *E. faecium* were susceptible to the same antibiotic. Both *vanA* and *vanH* genes were identified in all VRE isolates, although, no *vanB* gene was indicated. Homology analysis of sequenced amplicons verified the full length compatibility to the worldwide reported genes.

**Conclusion:**

The present study revealed VR *E.* *faecalis* in gastroenteritis patients and resistance factor for *vanA* and *vanH* genes are coordinated. Since enterococci isolates were all multidrug resistance, increase in VR *E.* *faecalis *vanA / vanH in this area could be expected.

## Introduction

 Enterococci are gram-positive, facultative anaerobic, oxidase- and catalase-negative, non-spore-forming cocci which usually inhabit the alimentary tract of mammals (1). Among enterococcal species, *E. faecalis* (90-95%) and *E. faecium* (5-10%) are the most common ones in human infections (2). They can sustain to the hostile conditions and a range of environmental stress, including extreme temperature (5-65 ºC), as well as high NaCl concentration. In addition, two species of *E. faecalis* and *E. faecium* are able to survive in other stress conditions such as pH (4.5-10.0) (3). 

Despite its long clinical success (low pathogenic-bacteria), enterococci have been considered as an opportunistic pathogen which is detected in nosocomial infections. For instance, they cause the infections, including endocarditic, hepatobiliary sepsis, urine infections, neonatal sepsis, as well as infections of post-surgery (2). Therefore, the issue of enterococci resistance has received considerable critical attention.

Vancomycin is a glycopeptide antimicrobial drug which was introduced in the 1950s and produced by the soil bacteria *Streptomyces orientalis *(4). It is active against most gram-positive bacteria, whereas the majority of gram negatives are resistant (5, 6). This antibiotic was mainly concerned for the clinical proposes in 1972 and vancomycin-resistant enterococci were reported for the first time 15 years later. Recent trends in the field of glycopeptides resistance such as vancomycin, teicoplanin, and aminoglycosides have led to the several studies (6). Moreover, a report conducted by CDC’s National Nosocomial Infections Surveillance (NNIS) in 1983-1989 showed 7.6% resistant enhancement (7). It has been objected as a drug of ‘last resort’ and has been classified as critically important for human medicine for treatment of patients with severe infections with multi-drug resistant *Enterococcus* spp. and meticillin resistant *Staphylococcus aureus* (MRSA). It is useful for intestinal infections due to the meager (poor) absorption of vancomycin when administered orally, especially in pseudomembranous colitis caused by *Clostridium difficile* (5).

The species of *E. faecalis* and *E. faecium* are notably recognized in human beings (8). Along with the increment in mortality and morbidity rate of these species (60%) as well as 75% death risk through vancomycin-resistant enterococci (VRE) (9); the VRE have been extensively concerned as a serious factor in the public health. Consequently, the occurrence of enterococci glycopeptides-resistant (GRE) in a patient leads to the fail of treatment along with death enhancement (10, 11). 

Recent developments in enterococci resistance indicated the involvement of genetic elements, including *vanA*, *B*, *C*, *D*, *E*, *G*, *L*, *M* and *N* from which the variants of *vanA*, *B* and *C* are the most prevalent in clinically relevant isolated species (8, 12). The variant *vanH* is one of the seven genes that form a gene cluster of *vanA* (13). The *vanA* and *vanB* resistance operons are chromosomally located and are able to transfer on the plasmid (14), which are usually associated with *E. faecalis* and *E. faecium* strains (15). 

The emergence of the first cases of VRE in the world has been expanding to such an extent that today it has become one of the most important causes of Hospital infections. VanA is known as the most common variant of the human resistance in most parts of the world, while in some countries such as Australia and Sweden, the majority of VRE cases were possessed vanB (16).

A considerable amount of literature has been published on vancomycin resistance in Iran. The species of *E. faecalis* and its resistance were identified with 3.6% frequency in hospitalized patients (17). Also, other researches from 2009-2010 revealed the prevalence of the same species (16%) at Children's Medical Center, in which all isolates were positive for the *vanA* gene (18).

The increase in antibiotic resistant enterococci worldwide and recent reports of the VRE presence in IRAN indicates the need for more epidemiological information. This research was conducted to study *vanA*, *vanB* and *vanH* genes which play the key role in resistance by means of PCR method in the isolates of vancomycin-resistant enterococci from gastrointestinal tract infection patients. 

## Patients and Methods

This cross-sectional study was performed in the Khatamolanbia Hospital of Shahrood (a city in the central region of Iran) from January to July 2014 among 265 intestinal infection patients. The fecal and rectal swab specimens were collected from outpatients and hospitalized patients, respectively. 

***Enterococci*** **isolation and Identification**

Isolation and identification of enterococci were carried out using specific methods. Initially, all samples were cultured in Kanamycin Esculin Azide agar (Merck, Germany) and incubated at 37°C for 48 h. Then, the black colonies were examined to certify the gram-positive cocci through gram staining technique. 

To identify the enterococci species, the biochemical tests, including catalase reaction, growth at 44°C and its tolerance in 6.5% NaCl, the presence of L-pyrrolidonyl-beta-naphthylamide (PYR), as well as the hydrolysis of esculin in the presence of bile were performed on the gram-positive colonies. In addition, the phenotypes of enterococcal isolates (*E. faecalis, and E. faecium*) were identified using the following experiments: glucose fermentation, D-mannitol, acid production of L-arabinose.

**Antibiogram (Kirby–Bauer antibiotic test)**

Assessment of determining susceptibility to antimicrobial was performed by Kirby-Bauer agar disk diffusion assay. The susceptibility of enterococci was determined for the following antibiotics; vancomycin (30 µg), kanamycin (30 µg), streptomycin (30 µg), erythromycin (15 µg), amikacin (30 µg), ampicilin (10 µg), gentamicin (120 µg), ciprofloxacin (5 µg), and choloramphenicol (30 µg) (Padtan Teb, Iran). To estimate the sensitivity of isolates to antibiotics, the size of inhibition zone was measured and compared against a reference standard chart of the Clinical and Laboratory Standards Institute (CLSI) (www.clsi.org)(19).

**Table 1:** Primer sequences used in polymerase chain reaction (PCR

**Reference**	**Amplicon size (bp)**	**T**_m_ ** (** ^o^ **C)**	**Primer sequence (5’ to 3’)**	**Primer name**	**Gene and accession No.**
[20]	732	59.0	GGGAAAACGACAATTGC	VanAF	*vanA* KM235680
		60.7	GTACAATGCGGCCGTA	VanAR	
[21]	536	61.9	AAGCTATGCAAGAAGCCATG	VanBF	*vanB* AY665550
		61.9	CCGACAATCAAATCATCCTC	VanBR	
GneBank AB663321	503	64.4	GTGAGCAGGATGAGGCAGA	vanHF	*vanH* KM235681
		63.5	GCTGCGACTATAAGCCAACAC	vanHR	

**Polymerase Chain Reaction (PCR)**

Genetic evaluation of resistance and determining the presence of *vanA*, *vanB*, and *vanH* variants were achieved by PCR method. Briefly, some colonies were resuspended in 1 mL of deionized water and boiled for 10 min. Subsequently, the extracted genomic DNA was centrifuged at 10000 rpm for 5 min. The reaction mixture including 2.5 µl of 10X PCR buffer, 0.4 mM dNTP, 1.5 mM MgCl_2_, 1 Unit Tag DNA polymerase, and 0.5 µM of both forward and reverse primers for the final volume of 25 µl. The PCR products were analyzed by 1% agarose gel. Table 1 indicates the features of primer sequences which were designed based on DNA sequences of target genes in GenBank.

**Cloning and Sequencing:**

All PCR DNA products were extracted and cloned into the pTZ57R vector (Fermentas, Lithuania) based on TA cloning scheme according to the manufacturer's directions. Ligation reaction was prepared in 1:3 molar ratio, including 3 µl pTZ57R plasmid, 5 µl PCR product, 1 µl T4 DNA ligase enzyme, 5 µl 5X buffer and 6 µl nuclease free distilled water. The mixture was incubated at 4 °C for 16 hours. The resulting plasmid was transformed into the *E. coli* strain DH5-α competent cells. Consequently, the cloned fragments were sequenced and then were analyzed in Blast tool of the NCBI server (http://blast.ncbi.nlm.nih.gov/Blast.cgi). 

## Results

**Figure 1:**
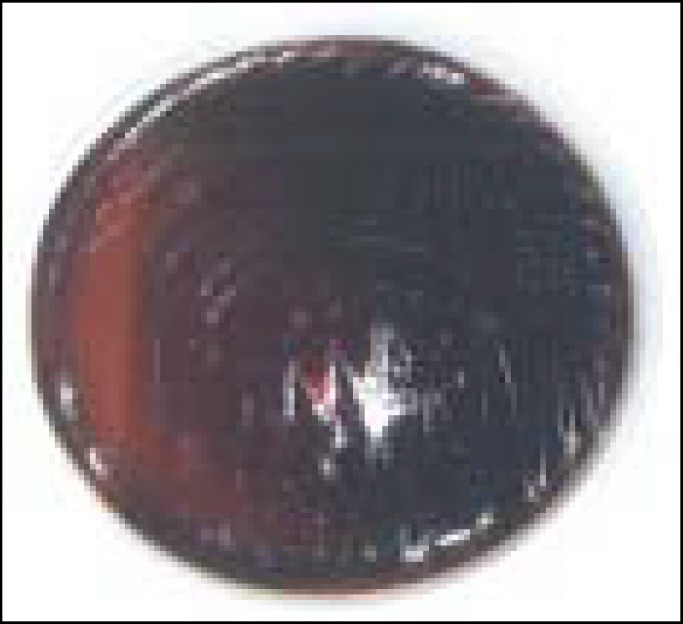
Growth of enterococci as black colonies on kanamycin Aesculin Azide agar

To assess the VRE isolates among 265 outpatients and hospitalized patients, fecal samples were cultured in kanamycin Aesculin Azide agar. The enterococci were grown in black colonies (Figure 1). The biochemical tests indicated that among all samples, 100 VRE isolates (~ 38%) were obtained. The *E. faecalis* and *E. faecium* isolates were presented in 91 (91%) and 9 (9%) cases, respectively. 

**Figure 2:**
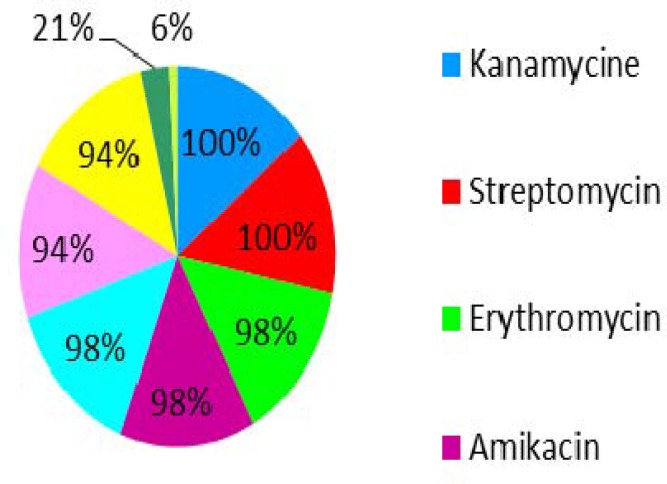
Percentage of resistant enterococci to different antibiotics

The results of the antibiotic sensitivity experiment are summarized in pie chart (Figure 2). It is apparent from this chart that the isolates were resistant 94% to 100% to seven antibiotics, including kanamycin, streptomycin, erythromycin, amikacin, ampicilin, gentamycin, and ciprofloxacin. The vancomycin and chloramphenicol resistant Enterococci isolates composed of 6% and 21% of cases, respectively. All vancomycin resistant isolates belong to *E. faecalis*. However, the *E. faecium* isolates were susceptible to the same antibiotic.

**Figure 3:**
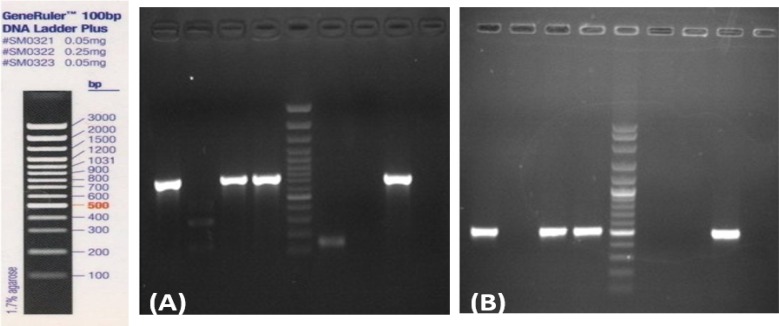
Polymerase chain reaction (PCR) to amplify the vancomycin resistant genes in vancomycin-resistant isolates in some patients. **(A)** Amplification of *vanA* gene (732 bp) in isolated VR *E. faecalis* from patients (lanes 1, 3, and 4); lane 2: isolated vancomycin-sensitive enterococci from patient; lanes 6 and 7 are negative and positive controls, respectively. **(B)** Amplification of *vanH* gene (503 bp) in the same patients of gel A. Lane 5: 100 bp DNA Ladder (Fermentas, USA

Figure 3 illustrates the experimental data on amplification of *vanA* and *vanH* genes in the entire vancomycin resistant isolates. However, the *vanB* resistant gene was not observed. As expected, the *vanA* amplified a 732-bp fragment and *vanH* yielded a 503-bp product. The accuracy of PCR reactions was verified with positive and negative controls.

The sequencing results of PCR products were analyzed in the Blast tool of NCBI server. The nucleotide sequences of *vanA* (KM235680) gene showed 100% identities with other *vanA* sequence data reported in the GenBank. Moreover, the blast sequence analysis of *vanH* (KM235681) gene revealed the same result. However, no *vanB* gene was identified (data not shown). 

## Discussion

Enterococci were known as normal flora of the gastrointestinal tract in humans and animals. However, in recent years, they have been considered as an opportunistic pathogen, particularly in nosocomial infections with elevated mortality and morbidity rate (9). The epidemiological studies indicated that enterococci are the second factor after *Staphylococcus aureus* for hospital infections in Iran, some of which are involved in peritonitis (100%), urinary tract infection (UTI, 50%), and more than 20% in Pneumonia, surgical site infection, as well as bloodstream infection (22).

As very little literature was found on the frequency of VRE in Iran, the current study was set out to assess the VRE frequency and the association of *vanA*, *vanB*, and *vanH* genes in patients with intestinal infection in a university hospital (Shahrood, the central region of Iran). 

The present research revealed that the enterococci were isolated in 38% of gastrointestinal tract infection patients. It should be noted, albeit, the reasons of VREs colonization was not the current research objective, but the high percentage of VRE obviously isolated from patients with gastrointestinal symptoms. The biochemical experiments-associated with identification of isolates were signified 91% in *E. faecalis* and 9% in *E. faecium*. The present findings seem to be consistent with other researches in Europe and United State which introduced the *faecalis* and afterward *faecium* as common species isolated from clinical specimens of patients with approximately 1:10 ratio (2, 23, 24). A previous study has also demonstrated the frequency of *E. faecalis* and *E. faecium* species in 57% and 43%, in UTI, wound and blood clinical samples in Iran, respectively (25).

Moreover, enterococci resistance to vancomycin and other glycopeptide antibiotics has been widely reported in many countries, in this respect, the elevated occurrence of VRE is catastrophic in recent years (7, 26, 27). The consequence of this study shows that all isolates were resistant to the referred antibiotics in the range of 94-100% except for choloramphenicol and vancomycin. Increased susceptibility of isolates to choloramphenicol in this study corroborates the earlier findings, which suggested that this finding is due to the limited usage of choloramphenicol and tetracycline antibiotics (28). It is surprising that only 6% of enterococci isolates were resistant to vancomycin. Furthermore, all vancomycin-resistant isolates were belonged to *E. faecalis *species; on the other hand, none of the *E. faecium *isolates were resistant to vancomycin.

These findings further support and suggest the idea of vancomycin as a critical antibiotic in the treatment of enterococci’s infections. In accordance with the present results, previous study has demonstrated that the VRE incidence was 3.6% in the northeast of Iran (17). However, other researches revealed the rate of VRE was 16% to 40% in urinary tract infection, injury and blood isolates (18, 25). Whereas, others were pointed out 22% VRE rate in fecal samples of hemodialysis patients (29). Therefore, there is a definite need for clinical treatment of VRE infections.

From the other point of view, the genotyping-associated with vancomycin resistance are known to be acquired by various *Enterococcus* spp. (30). The *vanA* and v*anB* are widespread amongst enterococci isolates. 

The *VanA* is characterized by high-level inducible resistance to vancomycin antibiotic (31). The gel examination of the PCR products proved that all vancomycin resistant isolates possess the *VanA* and *VanH* variants. Also, the blast analysis of sequencing outcomes was 100% compatible with the reported sequences of *vanA* (KM235680) and *vanH* (KM235681). Contrary to expectations, this study did not find the *vanB* genotype in any isolates. The *vanA* as a common genotype was reported in VRE isolates of Iranian patients as this genotype was recognized in isolated enterococci of urine infections in Iran (18). Another investigation showed the genotype of *vanA* in 90% of VREs strains regardless of enterococci species (32). However, another study in north-east of Iran indicated the *vanB* and *vanA* genotypes in species of *E. faecalis* and *E. faecium*, correspondingly (25). Unlike some countries such as Singapore, where the emergence of resistance is supplied by *vanB*, the resistance of VRE isolates in Iran is associated with both *vanA* and *vanB*. Therefore, it is likely that such connections exist between the ratios of *E. faecalis* to *E. faecium* species, value of vancomycin resistance and the diversity of genotypes such as *vanA* and *vanB*, which are dependent to the type of the clinical samples and geographical location. 

Overall, this research showed that most of the enterococci isolates from fecal samples of intestinal infection patients in the central region of Iran belonged to *E. faecalis *species; which are resistant to the majority of antibiotics. The VRE isolates are possible to be separated and identified; however, their frequencies are not high enough. The most obvious finding emerged from this investigation is that the antibiotic-resistant *E. faecalis *isolates carry both *vanA* and *vanH* in concert manner. Since *vanA* provides high-level resistance to vancomycin and increased transmission power to *vanB*; the enhancement of VR *E. faecalis VanA/VanH* in this area could be expected. 

In conclusion, based on this study, molecular genotype of VRE in Iran was identified, which could be supportive and suitable in identification of resistance pattern against this antibiotic, vancomycin, through the worldwide.
